# Integrating a web-based intervention into routine care of binge-eating disorder: Study protocol for a randomized controlled trial

**DOI:** 10.1016/j.invent.2022.100514

**Published:** 2022-02-21

**Authors:** Luise Pruessner, Steffen Hartmann, Julian A. Rubel, Christopher Lalk, Sven Barnow, Christina Timm

**Affiliations:** a Department of Psychology, Heidelberg University, Hauptstr. 47-51, 69117 Heidelberg, Germany; b Psychotherapy Research Unit, University of Giessen, Otto-Behaghel-Straße 10, 35394 Giessen, Germany

**Keywords:** Binge eating disorder, Web-based interventions, Randomized controlled trial, Ecological momentary assessment, Emotion regulation, Internet-based self-help

## Abstract

**Background:**

Although binge eating disorder (BED) is the most common eating pathology and carries a high mental and physical burden, access to specialized treatment is limited due to patient-related barriers and insufficient healthcare resources. Integrating web-based self-help programs into clinical care for BED may address this treatment gap by making evidence-based eating disorder interventions more accessible.

**Methods:**

A two-armed randomized controlled trial will be conducted to evaluate the effectiveness of a web-based self-help intervention for BED in routine care settings. Patients aged 18–65 years fulfilling the diagnostic criteria for BED (*N* = 152) will be randomly allocated to (1) an intervention group receiving a 12-week web-based self-help program or (2) a waitlist control group with delayed access to the intervention. The primary outcome will be the number of binge eating episodes. Secondary outcomes include global eating pathology, functional impairments, work capacity, well-being, comorbid psychopathology, self-esteem, and emotion regulation abilities. Measurements will be conducted at baseline (study entrance), 6 weeks after baseline (mid-treatment), and 12 weeks after baseline (post-treatment). To capture outcomes and treatment mechanisms in real-time, traditional self-reports will be combined with weekly symptom monitoring and ecological momentary assessment.

**Discussion:**

Evaluating the effectiveness of web-based interventions is essential to overcome the treatment gap for patients with BED. When adequately integrated into standard care, these programs have the potential to alleviate the high burden of BED for individuals, their families, and society.

**Trial registration:**

*https://clinicaltrials.gov/ct2/show/NCT04876183*, Identifier: NCT04876183 (registered on May 6th, 2021).

## Highlights

• Binge eating disorder is highly prevalent and associated with severe personal and societal costs as well as increased mortality.
• However, less than one in six individuals meeting the diagnostic criteria receive treatment, increasing the chronicity and the burden of illness.
• Integrating web-based programs into standard care could address this treatment gap and reduce the burden of binge eating disorder.
• To provide low-threshold access to treatment for binge eating disorders, we will test the effectiveness of a web-based intervention in routine care settings.
• Weekly reports and ecological momentary assessment will complement retrospective measures to obtain naturalistic eating pathology assessments with a high temporal resolution.

## 1 Introduction

### 1.1 Background and rationale

Binge eating disorder (BED) is characterized by recurrent episodes of consuming large amounts of food marked by a loss of control (APA, 2013) and is associated with critical impairments in physical health, social integration, professional performance, and overall quality of life (Ágh et al., 2015; Erskine et al., 2016; Udo and Grilo, 2020). Across the spectrum of eating disorders, BED is the most common, with a lifetime prevalence of approximately 2% for women and 1% for men (Cossrow et al., 2016; Erskine and Whiteford, 2018, Keski-Rahkonen, 2021). Typically co-occurring physical illnesses include obesity, hypertension, and type 2 diabetes (McCuen-Wurst et al., 2018; Udo and Grilo, 2019). Left untreated, BED often takes a chronic course (Pope et al., 2006; Udo and Grilo, 2018), and mortality rates are increased (Fichter and Quadflieg, 2016; Smink et al., 2012). In a representative sample in the United States, up to 23% of individuals with BED endorsed previous suicide attempts (Udo et al., 2019), and the majority (94%) fulfilled the criteria for at least one comorbid psychiatric disorder (Udo and Grilo, 2019). Based on these illness-related personal and societal costs, identifying and implementing effective BED treatments is fundamental.

Cognitive Behavioral Therapy (CBT) is the most established evidence-based intervention for BED (for meta-analyses, see Brownley et al., 2016; Hilbert et al., 2019; Linardon et al., 2018b), recommended by national treatment guidelines of the *National Institute for Health and Care Excellence* (NICE, 2017) and the *Association of Scientific Medical Societies in Germany* (Herpertz and Herpertz-Dahlmann, 2017). However, despite the effectiveness of CBT, treatment rates for BED are lower than those for many other mental disorders (Kessler et al., 2013; Silén et al., 2021). Specifically, only 49% of the patients fulfilling the criteria for BED are recognized in healthcare (Coffino et al., 2019), and of these, only 15–17% receive evidence-based treatments (Layard et al., 2012; Silén et al., 2021), increasing the risk of chronicity and the burden of illness (Striegel Weissman and Rosselli, 2017).

Barriers in the help-seeking process can explain this unmet need for treatment (Erskine et al., 2016; Linardon et al., 2021; Striegel Weissman and Rosselli, 2017). On the patient's side, feelings of shame and guilt, low change motivation, or lack of knowledge regarding eating disorders can prevent seeking professional help (Becker et al., 2010, Coffino et al., 2019). Contributing to these barriers, the severity of BED is publicly underestimated, and binge eating is frequently attributed to low self-discipline, increasing the stigma of the condition (Puhl and Suh, 2015). Service-related barriers are costs of mental health treatments, policy and legal constraints (e.g., restrictions regarding reimbursement), and limited availability or accessibility of evidence-based care (Kazdin et al., 2017). For example, in Germany, patients have to wait 20 weeks for psychotherapeutic outpatient treatment (BPtK, 2018). This delay is often longer for BED compared to other eating disorders, possibly due to a lack of awareness of this diagnostic category (Kessler et al., 2013, Kornstein et al., 2016). Consequently, improving access to specialized treatment is essential to reducing the burden and chronicity of BED.

Web-based self-help interventions can help overcome these barriers by making treatments for BED more available (Aardoom et al., 2013; Dölemeyer et al., 2013; Venkatesh et al., 2021). Empirical evidence suggests that web-based interventions can effectively reduce BED symptoms with medium to large effect sizes when investigating symptom changes after completing online self-help programs (e.g., Beintner et al., 2014; de Zwaan et al., 2017; Haderlein, 2022; Wyssen et al., 2021). Moreover, web-based self-help interventions have several advantages compared to face-to-face psychotherapy. They are cost-effective, easy to implement, have a low threshold, and are permanently accessible while allowing a flexible treatment adaptation based on individualized therapy goals (Aardoom et al., 2013; Dölemeyer et al., 2013; Linardon et al., 2020). Therefore, online programs for BED could be used to bridge waiting times for face-to-face therapy, facilitate transfers from inpatient to outpatient care, or may serve as an alternative treatment for patients who perceive the barriers of face-to-face therapy as too high (Beintner et al., 2014; Venkatesh et al., 2021).

Building on these advantages and addressing the need for more accessible evidence-based treatments for BED, the present two-armed randomized controlled trial will test the effectiveness of a web-based self-help intervention for patients with BED regarding reductions in core eating disorder symptoms and improvements in quality of life. As there is still limited knowledge of the course and outcome of BED compared to other eating disorders such as anorexia nervosa or bulimia nervosa (Kazdin et al., 2017, Smink et al., 2013), the current study adds to the existing BED treatment literature by evaluating a web-based intervention in standard care settings compared to a waitlist control condition. These findings address a significant research gap concerning the use and effects of web-based interventions under routine care settings, which remain largely understudied (Vollert et al., 2019). Furthermore, our study will test potential mechanisms of treatment success and predictors of intervention outcomes, such as changes in emotion regulation (Dingemans et al., 2017; Izadpanah et al., 2019), to understand which patients benefit most from online self-help interventions for BED. Finally, traditional self-report questionnaires will be complemented by ecological momentary assessment (EMA) using mobile technology to capture treatment outcomes and mechanisms in the natural environment and expand the ecological validity of our data to real-time experience (Munsch et al., 2009; Pruessner et al., 2021; Shiffman et al., 2008).

## 2 Methods

### 2.1 Objectives and hypotheses

The overarching goal of our trial is to evaluate the 12-week web-based self-help intervention *Selfapy* for BED, which employs CBT methods targeting binge eating pathology directly (Munsch, 2003; Munsch, 2007), as well as processes associated with the maintenance of BED, such as emotion regulation, stress management, and self-esteem (Dingemans et al., 2017, Linardon et al., 2019; Sipos and Schweiger, 2016). The intervention can be used via desktop browsers and mobile devices and has been established alongside a program targeting the treatment of bulimia nervosa in routine care settings (for details, see Hartmann et al., 2022).

Based on the effectiveness of online interventions for BED (Beintner et al., 2014; Haderlein, 2022), we expect that the program will lead to a greater reduction in the frequency of binge eating episodes over the twelve weeks of treatment compared to a waitlist control condition. Moreover, we assume that there will be a higher decline in global eating disorder symptoms and functional impairments as well as a higher increase in well-being and work capacity in the intervention group compared to the waitlist control group (Ágh et al., 2015, Jenkins et al., 2021, Safi et al., 2022). Finally, we expect that the web-based intervention for BED will be associated with a significantly higher reduction in comorbid psychopathology, increased self-esteem, and an improved ability to regulate negative emotions after treatment (Dingemans et al., 2017, Linardon et al., 2019, Prefit et al., 2019).

### 2.2 Participants and recruitment

Participants will be recruited via the intervention provider's website (https://www.selfapy.de), social media, mailing lists, self-help forums, a waitlist of subjects interested in the intervention, and information brochures distributed in various inpatient and outpatient treatment centers in Germany. Recruitment will be conducted in parallel with another study at Heidelberg University testing the effectiveness of a web-based intervention for bulimia nervosa (Hartmann et al., 2022). Individuals interested in participating can register online to receive detailed information about the procedure, complete an eligibility screening and schedule a clinical interview to assess the inclusion and exclusion criteria. Informed consent will be obtained from all participants, and subjects can ask questions about the study procedure. Participants included in the study will be reimbursed 30€ upon completing all study assessments (baseline, mid-treatment, post-treatment). The participant characteristics based on the PICO framework (Schardt et al., 2007) are depicted in the supplementary material (Table S1).

#### 2.2.1 Eligibility criteria

Inclusion criteria are (1) age between 18 and 65 years, (2) adequate German-language skills (C1), (3) having a smartphone with permanent internet access during the study period, and (4) meeting the diagnostic criteria for BED according to the fifth edition of the *Diagnostic and Statistical Manual of Mental Disorders* (DSM-5; APA, 2013). We will exclude individuals with (1) a Body Mass Index (BMI) below 18.5, (2) current psychotherapy or pharmacotherapy for eating disorders, (3) anorexia nervosa or bulimia nervosa, (4) comorbid bipolar disorder or psychotic disorders, (5) acute substance dependence, (6) current severe depressive episodes, and (7) acute suicidality. These comorbidities were selected as exclusion criteria as they may represent contraindications of using web-based self-help interventions (e.g., von Brachel et al., 2014, Wilson and Zandberg, 2012). Patients who do not meet the inclusion criteria due to their condition's severity are encouraged to seek professional help and are referred to alternative treatments. A primary diagnosis other than BED and other comorbid diagnoses are not exclusionary to best represent routine care. Subjects who meet the criteria for bulimia nervosa according to the DSM-5 will be included in our parallel study evaluating a web-based intervention for this condition (Hartmann et al., 2022). Patients receiving psychotherapy or pharmacological treatment for eating disorders at baseline are excluded, as changes in the primary and secondary endpoints cannot be attributed to the intervention in case of systematic pre-treatment group differences regarding healthcare services utilization. To maximize our findings' external validity and generalizability and best represent routine care in Germany, all participants are free to seek other health care services after randomization, including pharmacological and psychological treatments, which will be assessed throughout the trial.

### 2.3 Trial design

**Fig. 1:**
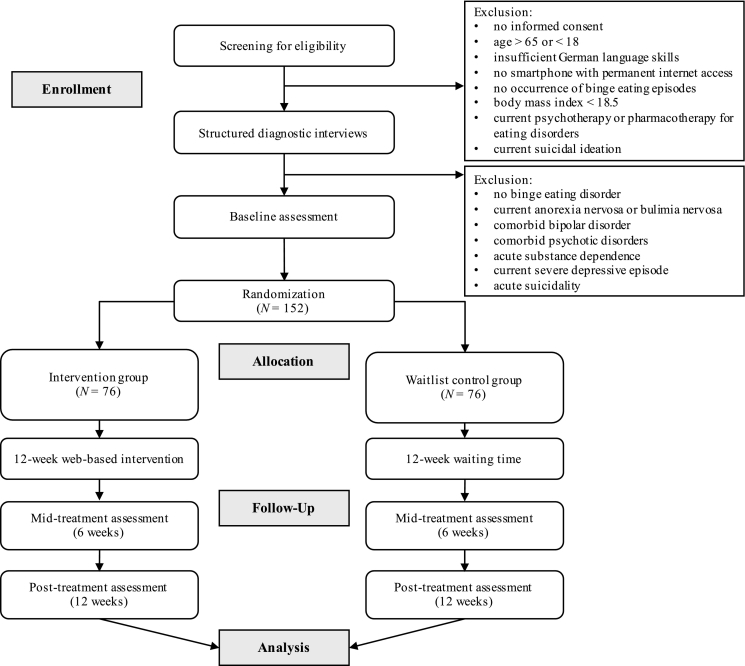
CONSORT flow of participants randomly assigned to a web-based intervention for binge eating disorder or a waitlist control condition.

A two-armed randomized controlled trial will be conducted to evaluate the effectiveness of the web-based intervention for BED. The CONSORT flow diagram (Altman and Schulz, 2001) for the study is presented in Fig. 1. Subjects who meet the inclusion criteria based on an eligibility screening and a diagnostic interview will be randomly allocated either to (1) an intervention group receiving immediate access to the web-based intervention for BED or (2) a waitlist control group with delayed access to the intervention (12 weeks). Assessments will be conducted at baseline (study entrance), 6 weeks after baseline (mid-treatment), and 12 weeks after baseline (post-treatment).

#### 2.3.1 Randomization and blinding

After completing the diagnostic interview and the baseline assessment, randomization will be performed in a 1:1 ratio by an independent researcher who is not involved in the project using a computer-based algorithm provided by the software *SoSci Survey* (Leiner, 2021). The diagnostic interviews will thus be conducted blindly; that is, the clinical interviewers do not know what treatment a participant will be allocated to in case of inclusion (*allocation sequence concealment*; Altman and Schulz, 2001). During the clinical interviews, participants are told that the assigned waiting time varies randomly between 0 and 12 weeks to prevent frustration, dropout, or other biases in the control group. Following randomization, all participants receive an email with either a code to immediately access the intervention or information that they will receive a code in 12 weeks. Finally, the group allocation variable will be blinded to allow unbiased data analyses. For this purpose, an independent researcher will delete all information in the data set indicating the group membership.

#### 2.3.2 Intervention

Patients in the intervention group will receive immediate access to the 12-week web-based treatment for BED (*Selfapy*) which can be used via desktop or mobile browsers (Linardon et al., 2021). The intervention is derived from evidence-based CBT methods and exercises (Munsch, 2003; Munsch, 2007; Sipos and Schweiger, 2016) and was developed in several piloting phases incorporating the feedback of BED patients. Drawing on a general diathesis-stress model, the intervention aims at improving the participants' understanding of risk factors and their coping abilities. After introducing this model, each lesson includes informative texts, videos, audio files, and interactive exercises focused on a specific topic, such as *eating behavior*, *emotion regulation*, *stress management*, and *weight control*. Core exercises include eating protocols, behavioral analysis of binge eating episodes, and cognitive restructuring (Munsch, 2003; Munsch, 2007; Sipos and Schweiger, 2016). The participants work on the contents of the intervention individually. However, minimal guidance is given by a psychologist who tracks the participants' progress, sends reminders to start the program in cases of low engagement, provides crisis management, and answers questions concerning the exercises. Furthermore, the participants receive technical support via an integrated messenger function. Based on the assumption that individual resources and risk factors vary across participants, the program is personally adaptable, consisting of a core course with six mandatory modules, followed by six individually selectable specialization areas. All modules remain freely available to the users for a year. Table 1 gives an overview of the core course, areas of specialization, and the suggested 12-week treatment course.

**Table 1:** Content of the 12-week web-based intervention (Selfapy) for binge eating disorder.

	Module	Exercises
Core course		
1	*Goal-setting*	Describing binge eating behavior and setting personal goals concerning the intervention
2	*Psychoeducation*	Recognizing triggers and causes of binge eating, eating protocols
3	*Eating behavior*	Strategies to prevent binge eating episodes, short- and long- term consequences of binge eating
4	*Negative thoughts*	Cognitive restructuring, identifying and replacing automatic negative thoughts associated with binge eating, falsifying negative cognitions
5	*Emotion regulation*	Regulating negative emotions to prevent binge eating episodes, identifying emotions, training effective regulatory strategies
6	*Stress management*	Improving stress management, defusing stress-increasing thoughts, relaxation training to reduce binge eating episodes
Optional content		
7	*Self-esteem*	Training self-confidence and self-acceptance as an essential treatment target of binge eating disorder
8	*Resources*	Recognizing personal resources and strengths, discovering new sources of resilience, increasing positive activities
9	*Social environment*	Optimizing social support and strengthening social competencies
10	*Mindfulness*	Formal and informal mindfulness exercises, integrating mindfulness into everyday eating behavior
11	*Weight control*	Healthy diet and sufficient exercise to transfer therapeutic success to daily life
12	*Relapse prevention*	Relapse prevention strategies, goals for further practice, strategies to avoid future binge eating episodes

#### 2.3.3 Control group

Patients in the control group will not receive the web-based intervention for BED during the 12-weeks following randomization. To best represent routine care, they are free to seek other forms of professional help, including pharmacological and psychological treatments. All concurrently used healthcare services in the intervention and control group will be captured at each assessment (Roick et al., 2001). Subjects in the control group will receive the intervention after completing the last measurement.

### 2.4 Measures

#### 2.4.1 Eligibility screening

During the initial eligibility screening, age, weight, height, current psychotherapy or pharmacotherapy for eating disorders, having a smartphone with permanent internet access during the study period, the occurrence of core eating disorder behaviors (based on the *Patient Health Questionnaire*; Gräfe et al., 2004; Löwe et al., 2002), and the risk for suicidality will be assessed (using the *Ask* *Suicide*-*Screening Questions*; Horowitz et al., 2020). Individuals who do not meet the screening criteria receive information on other healthcare services or the possibility to participate in other studies.

#### 2.4.2 Structured clinical interviews

Subjects fulfilling the screening criteria will be contacted via telephone or video call by an independent and trained researcher from Heidelberg University who will administer the *Eating Disorders Examination Interview* (EDE; Hilbert et al., 2004) to assess the DSM-5 criteria for BED and exclude possible diagnoses of anorexia nervosa or bulimia nervosa. Moreover, to test for exclusionary comorbid diagnoses of severe depressive episodes, bipolar disorder, substance use disorders, acute suicidality, and psychotic disorders according to the DSM-5, the *Diagnostic Interview for Mental Disorders* (DIPS-OA, Margraf et al., 2017) will be conducted. Interrater reliability will be calculated by coding 20 clinical interviews by two different research team members. All diagnoses will be discussed within the research team, and a licensed psychotherapist will supervise all diagnostic interviews.

#### 2.4.3 Outcomes

Primary and secondary outcome measures will be assessed at baseline (study entrance), six weeks after baseline (mid-treatment), and 12 weeks after baseline (post-treatment). Moreover, to continuously evaluate core eating disorder symptoms, the occurrence of binge eating episodes and overeating will be monitored weekly, and traditional self-reports will be complemented by EMA using mobile technology to capture treatment outcomes and mechanisms in the natural environment and increase the ecological validity of our data. All measures will be collected using the computer software *SoSci Survey* (Leiner, 2021) and made available to participants at http://www.s2survey.net/. An overview of the clinical outcome measures based on the SPIRIT recommendations (Chan et al., 2013) is provided in Table 2.

**Table 2:** SPIRIT schedule of the randomized controlled trial.

	Study period				
	Enrollment	Allocation	Post-allocation		Close-out
		*Study entrance*	*Weekly*	*Mid-treatment*	*Post-treatment*
Timepoint	*−t*_*1*_	*t*_*1*_	*week*_*1*_*–week*_*12*_	*t*_*2*_	*t*_*3*_
Enrollment					
Eligibility screen	+				
Informed consent	+				
*Clinical interviews*					
Eating Disorder Examination Interview (EDE)	+				
Diagnostic Interview for Mental Disorders (DIPS-OA)	+				
*Allocation*		+			
Intervention					
12-week web-based intervention *Selfapy* for binge eating disorder			+	+	
12-week waiting time			+	+	
Assessments					
*Primary outcome*					
Number of Binge Eating Episodes (EDE-Q)		+		+	+
*Secondary confirmatory outcomes*					
Eating Disorder Examination Questionnaire (EDE-Q)		+		+	+
Weekly Binges Questionnaire(WBQ)		+	+	+	+
Clinical Impairment Assessment (CIA)		+		+	+
World Health Organization Well-Being Index (WHO-5)		+		+	+
*i*MTA Productivity Cost Questionnaire (*i*PCQ)		+		+	+
*Secondary exploratory outcomes*					
Patient Health Questionnaire Depression Scale (PHQ-9)		+		+	+
Generalized Anxiety Disorder Scale (GAD-7)		+		+	+
Rosenberg Self-Esteem Scale (RSES)		+		+	+
Difficulties in Emotion Regulation Scale (DERS)		+		+	+
Heidelberg Form for Emotion Regulation Strategies (HFERST)		+		+	+
Ecological Momentary Assessment (EMA)		+			+
*Other measures*					
Client Sociodemographic Service Receipt Inventory (CSSRI)		+		+	+
Negative Effects Questionnaire (NEQ)				+	+
Attitudes Towards Online Interventions (APOI)		+			+
Patients' Therapy Expectation and Evaluation Scale (PATHEV)		+		+	+

#### 2.4.4 Primary outcome

• Changes in the number of binge eating episodes: In line with meta-analytic evidence (Beintner et al., 2014, Haderlein, 2022), the frequency of binge-eating episodes within the previous 28 days will be examined as the primary outcome measure. The items are based on the *Eating Disorders Examination Questionnaire* (EDE-Q; Hilbert et al., 2007), employing the DSM-5 definition of binge eating episodes. Previous studies support the reliability of the EDE-Q when examining binge eating episodes in patients with BED (test-retest reliability = .84; Reas et al., 2006).

#### 2.4.5 Secondary confirmatory outcomes

• Changes in global eating psychopathology: Global eating psychopathology will be investigated as a secondary outcome using the total score of the EDE-Q (Hilbert et al., 2007), which is derived from the dimensions of *weight concern*, *shape concern*, *eating concern*, and *restraint*. The eating psychopathology dimensions are assessed with 22 items on a 7-point Likert scale and Cronbach's α ranges between .85 and .93 for the subscales and .97 for the total score (Hilbert et al., 2007).
• Changes in the weekly frequency of binge eating episodes, overeating, and regular eating: The *Weekly Binges Questionnaire* (WBQ; Munsch et al., 2007; Munsch et al., 2019) will be used as a continuous method to monitor the frequency of objective and subjective binge eating episodes and overeating as secondary outcomes of BED symptom alterations. Weekly text messages throughout the study period will remind participants to report the occurrence and rate the severity of each of these eating episodes as well as regular eating within the last week on an 11-point scale (Munsch et al., 2007; Munsch et al., 2019).
• Changes in eating-disorder-related clinical impairments: To assess hypothesized reductions in clinical impairments specific to eating disorders, we will employ the *Clinical Impairment Assessment* scale (CIA; Bohn et al., 2008), which measures overall and domain-specific impairments (i.e., cognitive, social, and personal). The CIA consists of 16 items rated on a 7-point Likert scale and demonstrates excellent internal consistency (Cronbach's α = .97), construct validity, and sensitivity to change (Bohn et al., 2008).
• Changes in well-being: To measure assumed increases in well-being, we will use the frequently employed *World Health Organization-Five Well-Being Index* (WHO-5; Bech et al., 2003). The WHO-5 assesses general well-being based on five items, rated on a 6-point scale, and has high internal consistency (Cronbach's α = .92; Brähler et al., 2007).
• Changes in work capacity: To address the increasing significance of health economic evaluations (Jenkins et al., 2021; Safi et al., 2022), changes in work capacity and productivity will be measured based on the *iMTA Productivity Cost Questionnaire* (iPCQ; Bouwmans et al., 2015). The iPCQ examines absences from work and productivity losses due to sickness-related restrictions in work efficiency. Test-retest reliability of the iPCQ is excellent concerning the reported number of sick leave days (ICC = .83) and moderate regarding the number of days at work while impeded (ICC = .56) and efficiency rates (ICC = .73; Bouwmans et al., 2013).

#### 2.4.6 Secondary exploratory outcomes

• Changes in comorbid depressive symptoms: To capture a hypothesized decline in comorbid depressive symptoms, we will employ the *Patient Health Questionnaire-9* (PHQ-9; Kroenke et al., 2001). The PHQ-9 is a validated depression measure that assesses the severity of depressive symptoms on nine items using a 4-point Likert scale and has an internal consistency of Cronbach's α = .86 (Kroenke et al., 2001).
• Changes in comorbid anxiety symptoms: Changes in comorbid anxiety symptoms will be measured with the *General Anxiety Disorder Scale-7* (GAD-7; Löwe et al., 2008). The GAD-7 contains seven items answered on a 4-point scale and reliably measures generalized anxiety disorder symptoms (Cronbach's α = .89).
• Changes in self-esteem: Possible improvements in self-esteem will be assessed as a core treatment target of eating disorders (Linardon et al., 2019). To address this goal, the *Rosenberg Self-Esteem Scale* will be used (RSES; Roth et al., 2008), consisting of 10 items answered on a 4-point scale with an internal consistency of Cronbach's α = .88 (Roth et al., 2008).
• Changes in emotion regulation difficulties: Assumed decreases in emotion regulation difficulties following the intervention will be assessed using the *Difficulties in Emotion Regulation Scale* (DERS; Gratz and Roemer, 2004). The DERS consists of 36 items answered on a 5-point scale, and Cronbach's α ranges between .80 and .89 for the subscales and .93 for the total score.
• Use of emotion regulation strategies: The *Heidelberg Form for Emotion Regulation Strategies* (HFERST; Izadpanah et al., 2019) will be employed to assess the use of eight emotion regulation strategies (i.e., rumination, reappraisal, acceptance, problem solving, suppression of emotional expression, suppression of emotional experience, avoidance, social support) that have been associated with eating psychopathology (Prefit et al., 2019). The HFERST consists of 28 items answered on a 5-point scale, and Cronbach's α for the subscales ranges between α = .78 and α = .86 (Izadpanah et al., 2019).
• Ecological momentary assessment (EMA): An EMA protocol will be implemented on participants' mobile devices for five days at baseline and post-treatment using signal-contingent measurements at five random times each day and additional event-contingent assessments after episodes of binge eating (Munsch et al., 2007; Munsch et al., 2019; Schaefer et al., 2020). At every assessment, we will employ validated EMA items to measure affect (Watson and Clark, 1994), emotion regulation strategies (Izadpanah et al., 2019; Pruessner et al., 2021), and regulatory difficulties in daily life (Lavender et al., 2017). Binge eating episodes will be assessed based on the DSM-5 criteria for BED (APA, 2013; Munsch et al., 2009; Schaefer et al., 2020). Moreover, we will capture compensatory behavior, body image perceptions (Hilbert et al., 2007), and eating disorder urges using scales employed in previous intensive longitudinal designs (Tasca et al., 2009).

#### 2.4.7 Other measures

• Attitudes towards online interventions: Two subscales of the *Attitudes Towards Psychological Online Interventions Scale* (APOI; Schröder et al., 2015) will be used to assess attitudes towards web-based interventions. The selected subscales consist of eight items rated on a 5-point scale and reliably measure perceived technologization threat (α = .64) and anonymity benefits of online interventions (α = .62; Schröder et al., 2015).
• Patient outcome expectancies: Treatment motivation will be investigated using the *Patients' Therapy Expectation and Evaluation Scale* (PATHEV; Schulte, 2008). The PATHEV consists of 16 items answered on a 5-point scale and has been shown to reliably assess treatment motivation (α > .73; Schulte, 2005).
• Negative intervention effects: The *Negative Effects Questionnaire* (NEQ; Rozental et al., 2019) will be utilized to capture possible side effects of the intervention. For each of the 32 items, participants answer whether an adverse effect occurred (yes/no), how it was (0 to 4) and if they attribute the negative effect to the web-based intervention or something else. The NEQ has an excellent internal consistency of Cronbach's α = .95 (NEQ; Rozental et al., 2019).
• Use of other healthcare services: The *Client Sociodemographic Service Receipt Inventory – European Version* (CSSRI-EU; original: Chisholm et al., 2000; Roick et al., 2001) will be employed to assess the use of various other healthcare services (e.g., psychotherapy sessions, contact with psychiatrists). The CSSRI-EU has been validated as a reliable measure in clinical and non-clinical populations (Chisholm et al., 2000; Roick et al., 2001).
• Patient adherence: The log files on the online platform (*Selfapy*) will be utilized to capture patient adherence within the intervention group. This includes the times and dates participants log into the intervention and the number of completed modules. Moreover, after six and twelve weeks (mid- and post-treatment assessment), participants in the intervention group will be asked to report how frequently they accessed the web-based intervention.

### 2.5 Statistical methods

The analysis strategy for this trial consists of four steps: (1) descriptive analyses, (2) confirmatory analyses of the primary outcome, including sensitivity analyses, (3) analyses of secondary outcomes, including sensitivity analyses, (4) moderator and mediator analyses as well as analyses of the naturalistic EMA measures. All statistical tests will be conducted using R Statistics (R Core Team, 2020); see the R script in the supplementary materials (Supplement S2).

#### 2.5.1 Primary and secondary outcome analyses

To statistically evaluate the effectiveness of the web-based intervention for patients with BED compared to the waitlist control condition, growth models within a multilevel modeling (MLM) framework will be conducted. MLMs are regression-based models that allow considering the nested data structure, i.e., the three repeated assessments (level 1) nested within patients (level 2), define change as a continuous process, and have more power when handling missing data than traditional approaches (Hesser, 2015; Kahn and Schneider, 2013; Tasca and Gallop, 2009). To test whether there is a treatment × time interaction effect, we will set up MLMs of increasing complexity (Kahn and Schneider, 2013). The first model will have only random intercepts on the person-level (level 1), and no predictors will be included. In the second model, the fixed effects of time (study entrance, 6 weeks, 12 weeks) and treatment (intervention versus control group) will be added. In a final model, we will examine if the change in symptom severity differs between the intervention and control group by adding the treatment × time interaction. Model fit of the competing models will be compared employing likelihood ratio tests for nested models and the Akaike Information Criterion. A significant treatment × time-interaction with a more substantial change in participants undergoing the 12 weeks of treatment compared to the waitlist group will indicate confirmation of the hypotheses (Raudenbush and Bryk, 2002). The magnitude of treatment effects will be estimated as Cohen's *d* effect size (Feingold, 2013).

#### 2.5.2 Missing data and sensitivity analyses

As previous studies report notable dropout rates of web-based interventions (Linardon et al., 2018a), and the completer sample represents a subgroup of patients who may have particularly benefited from the intervention (Altman et al., 2001), both completer analyses and intent-to-treat analyses will be conducted. The treatment effects across the samples will be tested using two sensitivity analyses: (1) the conservative *last-observation-carried-forward* (LOCF) method, employing the last available measurement point of each subject, and (2) the *multiple imputations by chained equations* (MICE) approach using each participant's BMI, global eating psychopathology, number of binge-eating episodes determined in the EDE interview, and years since illness onset as predictors.

#### 2.5.3 Additional analyses

Additional statistical tests will be run to aid in interpreting the results and obtain a more nuanced understanding of the findings. Independent *t*-tests and χ^2^-tests will be used to estimate possible between-group pre-treatment differences regarding healthcare service utilization, demographic variables, and eating disorder symptom severity. In case of significant group differences, these variables will be utilized as moderators of the treatment × time interaction effects to test the robustness of the findings. We will further test whether other covariates such as patient outcome expectancies, attitudes towards online interventions, or patient adherence affect changes in the primary outcome. To obtain a higher ecological validity and temporal resolution when examining reductions in core eating disorder symptoms, we will analyze the EMA data and the weekly assessments of binge eating episodes. As patient safety indicators, the percentage of participants in the intervention group who experienced adverse intervention effects caused by the web-based intervention will be quantified, and the amount of impairment due to these negative effects will be calculated. Moreover, we will test potential improvements in emotion regulation difficulties and strategy use as possible mediators of BED symptom change.

### 2.6 Statistical power and sample size

To determine the required sample size, we conducted power calculations using the R package *powerlmm* (Magnusson, 2018). Based on previous meta-analytic evidence for the effectiveness of web-based interventions for BED (Beintner et al., 2014; Haderlein, 2022; Hilbert et al., 2019), we chose medium effect sizes of Cohen's *d* = 0.50 as a benchmark for expected pre-post differences between the intervention group and the waitlist control group. Power calculations using an intraclass correlation of .40 (Arend and Schäfer, 2019), a power of .80, and an alpha level of .05 resulted in a required sample of *N* = 152. Additional power analyses, including possible dropouts of 20% and different intra-class correlation coefficients, can be found in the supplementary material (Fig. S3), strengthening our conclusion that our sample will be sufficiently large to detect a medium effect under different statistical assumptions.

## 3 Discussion

BED is the most prevalent eating disorder and is associated with marked impairments in physical health, social integration, professional performance, and quality of life (Ágh et al., 2015; Erskine et al., 2016; Stice et al., 2009; Udo and Grilo, 2020). Nevertheless, treatment rates for BED are lower than for many other mental disorders, including eating disorders, such as anorexia nervosa and bulimia nervosa (Coffino et al., 2019, Hay et al., 2020, Kessler et al., 2013, Silén et al., 2021, Smink et al., 2013). Consequently, improving access to specialized treatment and decreasing barriers in the help-seeking process is an important goal to reduce the burden of illness and chronicity in BED.

Web-based interventions can reduce these barriers and facilitate access to evidence-based treatment for patients with BED (Aardoom et al., 2013; Dölemeyer et al., 2013; Venkatesh et al., 2021). However, despite the increasing number of empirical studies investigating web-based self-help for depression and anxiety symptoms (for a meta-analysis, see Spek et al., 2007), there is still a scarcity of high-quality studies on the effectiveness of online interventions for BED (Haderlein, 2022). Therefore, examining how and whether these programs successfully treat BED when implemented in health care systems remains an important avenue for future research. Building on previous research of technology-based eating disorder interventions (Beintner et al., 2014; Haderlein, 2022), the present study aims to evaluate the effectiveness of a web-based CBT intervention for BED in routine care settings.

### 3.1 Strengths and limitations of the study

We highlight five major strengths of this trial. First, the two-armed randomized controlled design provides a high degree of internal validity, allowing to attribute observed group differences to the web-based intervention with sufficient certainty. Second, the 12-week waiting period for the intervention was chosen to maximize our findings' generalizability and external validity. As such, a waiting time of three months closely reflects care reality for patients with BED in Germany, as awaiting outpatient psychotherapy requires, on average, three to six months (BPtK, 2018). Third, investigations of improvements in well-being and quality of life following web-based interventions remain scarce. To address this gap, we will assess crucial secondary outcome parameters such as changes in self-esteem and work capacity to evaluate whether web-based interventions can improve BED patients' overall daily life experiences apart from symptom reduction. Fourth, according to recent methodological recommendations (Hesser, 2015), our statistical analysis will use a linear mixed models framework. This approach has several advantages over traditional analysis methods, as it allows considering the nested data structure and has more power when handling missing data. Finally, to increase the validity of our findings, the structured diagnostic interviews will be conducted by independent researchers who are blind to treatment conditions. Moreover, biases associated with retrospective assessments will be reduced by closely tracking symptom fluctuations over the study period. To achieve this goal, we will combine classic self-report instruments with weekly reports of eating disorder symptoms (Munsch et al., 2007; Munsch et al., 2019) and naturalistic EMA to obtain a high temporal resolution and ecological validity of measures.

These strengths have to be set against the limitations. First, due to the relatively high dropout rates of web-based interventions (Linardon et al., 2018; Puls et al., 2020), low adherence might become a problem in the current study. This challenge may particularly apply to our trial design, as regular weekly assessments and intensive longitudinal measures collected in everyday life carry a higher participant burden. To increase motivation and adherence in both groups, participants will be reminded of the study using weekly text messages, regular emails, and phone calls. Moreover, recruiting a large clinical sample of participants fulfilling the criteria for BED is necessary to achieve adequate statistical power. However, BED is still under-recognized (Brownley et al., 2016, Coffino et al., 2019, Cossrow et al., 2016, Kazdin et al., 2017, Keski-Rahkonen, 2021, Silén et al., 2021) as it represents a relatively new diagnosis introduced in the DSM-5 (APA, 2013) and the eleventh revision of the International Classification of Diseases (ICD-11; Reed et al., 2019). Therefore, knowledge concerning the condition among healthcare providers and the general population remains scarce (Keski-Rahkonen, 2021; Venkatesh et al., 2021). To ensure the necessary sample size, we will allow participation from different countries (if subjects have sufficient German language skills) and employ a broad range of recruitment methods, including social media posts, email distributors, flyers in healthcare settings, and waitlists of patients with BED, which will enhance the chances of achieving the required sample size.

### 3.2 Conclusion

Bridging the gap between current knowledge about effective BED treatments and available interventions in clinical care is critical for advancing healthcare for this condition. The present randomized controlled trial aims to address this gap by testing whether providing low-threshold access to a web-based intervention for BED in routine care may help patients reduce their core symptomatology and improve their emotional and social well-being. Our findings will thus address the scarcity of studies evaluating the effectiveness of web-based interventions for eating disorders in standard healthcare settings (e.g., Vollert et al., 2019). Finally, to better understand which patients particularly benefit from web-based programs, our randomized controlled trial is designed to test individual treatment trajectories and mechanisms of change. Understanding for whom and why these interventions reduce eating disorder symptoms is essential to providing more targeted, ultimately more effective BED treatments. When successfully integrated into clinical practice, delivering the web-based self-help intervention to individuals in need of services may alleviate the high burden of BED for patients, their families, and society.

## Abbreviations

APA: American Psychiatric Association
APOI: Attitudes Towards Online Interventions
AZ: Aktenzeichen [File number]
BED: Binge Eating Disorder
BMI: Body Mass Index
BPtK: Bundespsychotherapeutenkammer [German national association of psychotherapists]
CBT: Cognitive-Behavioral Therapy
CIA: Clinical Impairment Assessment
CONSORT: Consolidated Standards of Reporting Trials
CSSRI: Client Sociodemographic Service Receipt Inventory
DERS: Difficulties in Emotion Regulation Scale
DSM-5: Diagnostic and Statistical Manual of Mental Disorders
DIPS: Diagnostic Interview for Mental Disorders
EDE: Eating Disorder Examination Interview
EDE-Q: Eating Disorder Examination Questionnaire
EMA: Ecological Momentary Assessment
EU: European Union
GAD-7: Generalized Anxiety Disorder Scale-7
HFERST: Heidelberg Form for Emotion Regulation Strategies
ICC: Intraclass Correlation Coefficient
ICD-11: International Classification of Diseases, Eleventh Revision
*i*PCQ: Productivity Cost Questionnaire
LOCF: Last Observation Carried Forward
MICE: Multiple Imputations by Chained Equations
MLM: Multilevel Modeling
*i*MTA: Institute for Medical Technology Assessment
NEQ: Negative Effects Questionnaire
PATHEV: Patients' Therapy Expectation and Evaluation Scale
PHQ-9: Patient Health Questionnaire-9
PICO: Population Intervention Compared Outcome
RSES: Rosenberg Self-Esteem Scale
SPIRIT: Standard Protocol Items: Recommendations for Interventional Trials
WBQ: Weekly Binges Questionnaire
WHO-5: World Health Organization-Five Well-Being Index

## Trial status

Recruitment started in January 2021 and is still ongoing. The first patient was enrolled in the study on January 15th, 2021. Assessments are expected to be completed by May 2022.

## Funding

The study will be funded by a European Regional Development Fund awarded to *Selfapy.* Publication fees will be financially supported by the *German Research Foundation* (Deutsche Forschungsgemeinschaft) Open Access Publishing Fund at Heidelberg University. The funders have no authority over the study design, collection, management, analysis, interpretation of data, writing of the report, and the decision to publish the findings.

## CRediT authorship contribution statement

LP and CT designed the study. SH and LP performed the sample size calculations and drafted the statistical design of the trial. LP and SH wrote the first draft of the manuscript. All co-authors (SH, JR, CL, SB, CT) contributed to critical revisions of the paper and approved the final manuscript.

## Ethics approval and consent to participate

Ethics approval has been obtained from the institutional review board at Heidelberg University (AZ Tim 2020 1/1). Informed consent will be obtained from all participants, and the trial will be conducted in compliance with the Declaration of Helsinki and good clinical practice. International data privacy regulations and EU legislation will be considered.

## Availability of data and material

The de-identified and anonymized data and the R analysis script of the current trial will be made available on the *Open Science Framework* (https://osf.io/pknbz/?view_only=9a0c786764bb4552b52be685a51220af).

## Declaration of competing interest

The authors declare that they have no known competing financial interests or personal relationships that could have appeared to influence the work reported in this paper.

## Appendix A Supplementary data

Supplementary material to the randomized controlled trial of a web-based intervention for binge eating disorder. Image 1

## References

[bb0005] Aardoom J.J., Dingemans A.E., Spinhoven P., Van Furth E.F. (2013). Treating eating disorders over the internet: a systematic review and future research directions. Int. J. Eat. Disord..

[bb0010] Ágh T., Kovács G., Pawaskar M., Supina D., Inotai A., Vokó Z. (2015). Epidemiology, health-related quality of life and economic burden of binge eating disorder: a systematic literature review. Eat. Weight Disord..

[bb0015] Altman D.G., Schulz K.F. (2001). Concealing treatment allocation in randomised trials. BMJ.

[bb0020] Altman D.G., Schulz K.F., Moher D., Egger M., Davidoff F., Elbourne D., Lang T. (2001). The revised CONSORT statement for reporting randomized trials: explanation and elaboration. Ann. Intern. Med..

[bb0025] APA (2013).

[bb0030] Arend M.G., Schäfer T. (2019). Statistical power in two-level models: a tutorial based on Monte Carlo simulation. Psychol. Methods.

[bb0040] Bech P., Olsen L.R., Kjoller M., Rasmussen N.K. (2003). Measuring well-being rather than the absence of distress symptoms: a comparison of the SF-36 mental health subscale and the WHO-five well-being scale. Int. J. Methods Psychiatr. Res..

[bb0045] Becker A.E., Hadley Arrindell A., Perloe A., Fay K., Striegel-Moore R.H. (2010). A qualitative study of perceived social barriers to care for eating disorders: perspectives from ethnically diverse health care consumers. Int. J. Eat. Disord..

[bb0050] Beintner I., Jacobi C., Schmidt U.H. (2014). Participation and outcome in manualized self-help for bulimia nervosa and binge eating disorder - a systematic review and metaregression analysis. Clin. Psychol. Rev..

[bb0055] Bohn K., Doll H.A., Cooper Z., O'Connor M., Palmer R.L., Fairburn C.G. (2008). The measurement of impairment due to eating disorder psychopathology. Behav. Res. Ther..

[bb0060] Bouwmans C., De Jong K., Timman R., Zijlstra-Vlasveld M., Van der Feltz-Cornelis C., Tan S.S., Hakkaart-van Roijen L. (2013). Feasibility, reliability and validity of a questionnaire on healthcare consumption and productivity loss in patients with a psychiatric disorder (TiC-P). BMC Health Serv. Res..

[bb0065] Bouwmans C., Krol M., Severens H., Koopmanschap M., Brouwer W., Hakkaart-van Roijen L. (2015). The iMTA productivity cost questionnaire: a standardized instrument for measuring and valuing health-related productivity losses. Value Health.

[bb0075] von Brachel R., Hötzel K., Hirschfeld G., Rieger E., Schmidt U., Kosfelder J., Vocks S. (2014). Internet-based motivation program for women with eating disorders: eating disorder pathology and depressive mood predict dropout. J. Med. Internet Res..

[bb0070] BPtK (2018). https://www.bptk.de/wp-content/uploads/2019/01/20180411_bptk_studie_wartezeiten_2018.pdf.

[bb0080] Brähler E., Mühlan H., Albani C., Schmidt S. (2007). Teststatistische Prüfung und Normierung der deutschen Versionen des EUROHIS-QOL Lebensqualität-Index und des WHO-5 Wohlbefindens-Index [Test statistical examination and norms of the German versions of the EUROHIS-QOL quality of life index and the WHO-5 well-being index]. Diagnostica.

[bb0085] Brownley K.A., Berkman N.D., Peat C.M., Lohr K.N., Cullen K.E., Bann C.M., Bulik C.M. (2016). Binge-eating disorder in adults: a systematic review and meta-analysis. Ann. Intern. Med..

[bb0090] Chan A.-W., Tetzlaff J.M., Altman D.G., Laupacis A., Gøtzsche P.C., Krleža-Jerić K., Berlin J.A. (2013). SPIRIT 2013 statement: defining standard protocol items for clinical trials. Ann. Intern. Med..

[bb0095] Chisholm D., Knapp M.R.J., Knudsen H.C., Amaddeo F., Gaite L., Van Wijngaarden B., Group E.S. (2000). Client socio-demographic and service receipt inventory–European version: development of an instrument for international research: EPSILON Study 5. Br. J. Psychiatry.

[bb9000] Coffino J.A., Udo T., Grilo C.M. (2019). Rates of help-seeking in US adults with lifetime DSM-5 eating disorders: prevalence across diagnoses and differences by sex and ethnicity/race. Mayo Clin. Proc..

[bb0105] Cossrow N., Pawaskar M., Witt E.A., Ming E.E., Victor T.W., Herman B.K., Erder M.H. (2016). Estimating the prevalence of binge eating disorder in a community sample from the United States: comparing DSM-IV-TR and DSM-5 criteria. J. Clin. Psychiatry.

[bb0110] Dingemans A., Danner U., Parks M. (2017). Emotion regulation in binge eating disorder: a review. Nutrients.

[bb0115] Dölemeyer R., Tietjen A., Kersting A., Wagner B. (2013). Internet-based interventions for eating disorders in adults: a systematic review. BMC Psychiatry.

[bb9010] Erskine H., Whiteford H.A. (2018). Epidemiology of binge eating disorder. Curr. Opin. Psychiatry.

[bb0125] Erskine H.E., Whiteford H.A., Pike K.M. (2016). The global burden of eating disorders. Curr. Opin. Psychiatry.

[bb0130] Feingold A. (2013). A regression framework for effect size assessments in longitudinal modeling of group differences. Rev. Gen. Psychol..

[bb0135] Fichter M.M., Quadflieg N. (2016). Mortality in eating disorders - results of a large prospective clinical longitudinal study. Int. J. Eat. Disord..

[bb0140] Gräfe K., Zipfel S., Herzog W., Löwe B. (2004). Screening psychischer Störungen mit dem gesundheitsfragebogen für patienten (PHQ-D). Diagnostica.

[bb0150] Gratz K.L., Roemer L. (2004). Multidimensional assessment of emotion regulation and dysregulation: development, factor structure, and initial validation of the difficulties in emotion regulation scale. J. Psychopathol. Behav. Assess..

[bb0155] Haderlein T.P. (2022). Efficacy of technology-based eating disorder treatment: A meta-analysis. Curr. Psychol..

[bb0160] Hartmann S., Pruessner L., Rubel J., Lalk C., Barnow S., Timm C. (2022). Applying a web-based self-help intervention for bulimia nervosa in routine care: study protocol for a randomized controlled trial. Internet Interv..

[bb0165] Hay P., Ghabrial B., Mannan H., Conti J., Gonzalez-Chica D., Stocks N., Touyz S. (2020). General practitioner and mental healthcare use in a community sample of people with diagnostic threshold symptoms of bulimia nervosa, binge-eating disorder, and other eating disorders. Int. J. Eat. Disord..

[bb0170] Herpertz S., Herpertz-Dahlmann B. (2017). S3-Leitlinien Diagnostik und Therapie der Essstörungen [S3-Guidelines for diagnostics and therapy of eating disorders]. Psychotherapeut.

[bb0175] Hesser H. (2015). Modeling individual differences in randomized experiments using growth models: recommendations for design, statistical analysis and reporting of results of internet interventions. Internet Interv..

[bb0180] Hilbert A., Tuschen-Caffier B., Ohms M. (2004). Eating Disorder Examination: Deutschsprachige Version des strukturierten Essstörungsinterviews [German version of the Eating Disorder Examination Interview]. Diagnostica.

[bb0185] Hilbert A., Tuschen-Caffier B., Karwautz A., Niederhofer H., Munsch S. (2007). Eating Disorder Examination-Questionnaire. Diagnostica.

[bb0190] Hilbert A., Petroff D., Herpertz S., Pietrowsky R., Tuschen-Caffier B., Vocks S., Schmidt R. (2019). Meta-analysis of the efficacy of psychological and medical treatments for binge-eating disorder. J. Consult. Clin. Psychol..

[bb0195] Horowitz L.M., Snyder D.J., Boudreaux E.D., He J.-P., Harrington C.J., Cai J., Chaves J.F. (2020). Validation of the ask suicide-screening questions for adult medical inpatients: a brief tool for all ages. Psychosomatics.

[bb0200] Izadpanah S., Barnow S., Neubauer A.B., Holl J. (2019). Development and validation of the Heidelberg form for emotion regulation strategies (HFERST): factor structure, reliability, and validity. Assessment.

[bb0205] Jenkins P.E., Luck A., Violato M., Robinson C., Fairburn C.G. (2021). Clinical and cost-effectiveness of two ways of delivering guided self-help for people with an eating disorder: a multi-arm randomized controlled trial. Int. J. Eat. Disord..

[bb0210] Kahn J.H., Schneider W.J. (2013). It's the destination and it's the journey: using multilevel modeling to assess patterns of change in psychotherapy. J. Clin. Psychol..

[bb0215] Kazdin A.E., Fitzsimmons-Craft E.E., Wilfley D.E. (2017). Addressing critical gaps in the treatment of eating disorders. Int. J. Eat. Disord..

[bb0220] Keski-Rahkonen A. (2021). Epidemiology of binge eating disorder: prevalence, course, comorbidity, and risk factors. Curr. Opin. Psychiatry.

[bb0225] Kessler R.C., Berglund P.A., Chiu W.T., Deitz A.C., Hudson J.I., Shahly V., Xavier M. (2013). The prevalence and correlates of binge eating disorder in the World Health Organization world mental health surveys. Biol. Psychiatry.

[bb9020] Kornstein S.G., Kunovac J.L., Herman B.K., Culpepper L. (2016). Recognizing binge-eating disorder in the clinical setting: a review of the literature. Prim. Care Companion CNS Disord..

[bb0230] Kroenke K., Spitzer R.L., Williams J.B. (2001). The PHQ-9: validity of a brief depression severity measure. J. Gen. Intern. Med..

[bb0235] Lavender J.M., Tull M.T., DiLillo D., Messman-Moore T., Gratz K.L. (2017). Development and validation of a state-based measure of emotion dysregulation: the state difficulties in emotion regulation scale (S-DERS). Assessment.

[bb0240] Layard R., Banerjee S., Bell S., Clark D., Fielding S., Knapp M., Scott S.V. (2012). http://eprints.lse.ac.uk/id/eprint/44572.

[bb0245] Leiner D.J. (2021). https://www.s2survey.net.

[bb0250] Linardon J., Hindle A., Brennan L. (2018). Dropout from cognitive-behavioral therapy for eating disorders: a meta-analysis of randomized, controlled trials. Int. J. Eat. Disord..

[bb0255] Linardon J., Messer M., Fuller-Tyszkiewicz M. (2018). Meta-analysis of the effects of cognitive-behavioral therapy for binge-eating–type disorders on abstinence rates in nonrandomized effectiveness studies: comparable outcomes to randomized, controlled trials?. Int. J. Eat. Disord..

[bb0260] Linardon J., Kothe E.J., Fuller-Tyszkiewicz M. (2019). Efficacy of psychotherapy for bulimia nervosa and binge-eating disorder on self-esteem improvement: meta-analysis. Eur. Eat. Disord. Rev..

[bb0265] Linardon J., Rosato J., Messer M. (2020). Break binge eating: reach, engagement, and user profile of an internet-based psychoeducational and self-help platform for eating disorders. Int. J. Eat. Disord..

[bb0270] Linardon J., Messer M., Lee S., Rosato J. (2021). Perspectives of e-health interventions for treating and preventing eating disorders: descriptive study of perceived advantages and barriers, help-seeking intentions, and preferred functionality. Eat. Weight Disord..

[bb0275] Löwe B., Spitzer R., Zipfel S., Herzog W. (2002). https://www.klinikum.uni-heidelberg.de/fileadmin/Psychosomatische_Klinik/download/PHQ_Manual1.pdf.

[bb0280] Löwe B., Decker O., Müller S., Brähler E., Schellberg D., Herzog W., Herzberg P.Y. (2008). Validation and standardization of the generalized anxiety disorder screener (GAD-7) in the general population. Med. Care.

[bb0285] Magnusson K. (2018). https://cran.r-project.org/package=powerlmm.

[bb0290] Margraf J., Cwik J., Suppiger A., Schneider S. (2017). http://dips-interviews.rub.de.

[bb0295] McCuen-Wurst C., Ruggieri M., Allison K.C. (2018). Disordered eating and obesity: associations between binge eating-disorder, night-eating syndrome, and weight-related co-morbidities. Ann. N. Y. Acad. Sci..

[bb0300] Munsch S. (2003).

[bb0305] Munsch S. (2007).

[bb0310] Munsch S., Biedert E., Meyer A., Michael T., Schlup B., Tuch A., Margraf J. (2007). A randomized comparison of cognitive behavioral therapy and behavioral weight loss treatment for overweight individuals with binge eating disorder. Int. J. Eat. Disord..

[bb0315] Munsch S., Meyer A.H., Milenkovic N., Schlup B., Margraf J., Wilhelm F.H. (2009). Ecological momentary assessment to evaluate cognitive-behavioral treatment for binge eating disorder. Int. J. Eat. Disord..

[bb0320] Munsch S., Wyssen A., Vanhulst P., Lalanne D., Steinemann S.T., Tuch A. (2019). Binge-eating disorder treatment goes online–feasibility, usability, and treatment outcome of an internet-based treatment for binge-eating disorder: study protocol for a three-arm randomized controlled trial including an immediate treatment, a waitlist, and a placebo control group. Trials.

[bb0325] NICE (2017). https://www.nice.org.uk/guidance/ng69.

[bb0330] Pope H.J., Harrison G., Lalonde J.K., Pindyck L.J., Walsh T., Bulik C.M., Crow S.J., Hudson J.I. (2006). Binge eating disorder: a stable syndrome. Am. J. Psychiatr..

[bb0335] Prefit A.-B., Cândea D.M., Szentagotai-Tătar A. (2019). Emotion regulation across eating pathology: a meta-analysis. Appetite.

[bb0340] Pruessner L., Hartmann S., Holt D.V., Schulze K., Barnow S. (2021). https://osf.io/md9ka/.

[bb0345] Puhl R., Suh Y. (2015). Stigma and eating and weight disorders. Curr. Psychiatry Rep..

[bb0350] Puls H.C., Schmidt R., Herpertz S., Zipfel S., Tuschen-Caffier B., Friederich H.C., Hilbert A. (2020). Adherence as a predictor of dropout in internet-based guided self-help for adults with binge-eating disorder and overweight or obesity. Int. J. Eat. Disord..

[bb0355] R Core Team (2020). R: a language and environment for statistical computing.

[bb0360] Raudenbush S.W., Bryk A.S. (2002).

[bb0365] Reas D.L., Grilo C.M., Masheb R.M. (2006). Reliability of the eating disorder examination-questionnaire in patients with binge eating disorder. Behav. Res. Ther..

[bb0370] Reed G.M., First M.B., Kogan C.S., Hyman S.E., Gureje O., Gaebel W., Tyrer P. (2019). Innovations and changes in the ICD-11 classification of mental, behavioural and neurodevelopmental disorders. World Psychiatry.

[bb0380] Roick C., Kilian R., Matschinger H., Bernert S., Mory C., Angermeyer M.C. (2001). Die deutsche Version des Client Sociodemographic and Service Receipt Inventory [The German version of the Client Sociodemographic and Service Receipt Inventory]. Psychiatr. Prax..

[bb0385] Roth M., Decker O., Herzberg P.Y., Brähler E. (2008). Dimensionality and norms of the Rosenberg self-esteem scale in a German general population sample. Eur. J. Psychol. Assess..

[bb0390] Rozental A., Kottorp A., Forsström D., Månsson K., Boettcher J., Andersson G., Carlbring P. (2019). The negative effects questionnaire: psychometric properties of an instrument for assessing negative effects in psychological treatments. Behav. Cogn. Psychother..

[bb0395] Safi F., Aniserowicz A.M., Colquhoun H., Stier J., Nowrouzi-Kia B. (2022). Impact of eating disorders on paid or unpaid work participation and performance: a systematic review and meta-analysis protocol. Int. J. Eat. Disord..

[bb0400] Schaefer L.M., Smith K.E., Anderson L.M., Cao L., Crosby R.D., Engel S.G., Wonderlich S.A. (2020). The role of affect in the maintenance of binge-eating disorder: evidence from an ecological momentary assessment study. J. Abnorm. Psychol..

[bb0405] Schardt C., Adams M.B., Owens T., Keitz S., Fontelo P. (2007). Utilization of the PICO framework to improve searching PubMed for clinical questions. BBMC Med. Inform. Decis. Mak..

[bb0410] Schröder J., Sautier L., Kriston L., Berger T., Meyer B., Späth C., Moritz S. (2015). Development of a questionnaire measuring attitudes towards psychological online Interventions–the APOI. J. Affect. Disord..

[bb9030] Schulte D. (2005). Messung der Therapieerwartung und Therapieevaluation von Patienten (PATHEV) [Measurement of patients’ expectation and evaluation of psychotherapy (PATHEV)]. Z. Klin. Psychol. Psychother..

[bb0415] Schulte D. (2008). Patients’ outcome expectancies and their impression of suitability as predictors of treatment outcome. Psychother. Res..

[bb0420] Shiffman S., Stone A.A., Hufford M.R. (2008). Ecological momentary assessment. Annu. Rev. Clin. Psychol..

[bb0425] Silén Y., Sipilä P.N., Raevuori A., Mustelin L., Marttunen M., Kaprio J., Keski-Rahkonen A. (2021). Detection, treatment, and course of eating disorders in Finland: a population-based study of adolescent and young adult females and males. Eur. Eat. Disord. Rev..

[bb0430] Sipos V., Schweiger U. (2016).

[bb0440] Smink F.R., Van Hoeken D., Hoek H.W. (2012). Epidemiology of eating disorders: incidence, prevalence and mortality rates. Curr. Psychiatry Rep..

[bb0445] Smink F.R., van Hoeken D., Hoek H.W. (2013). Epidemiology, course, and outcome of eating disorders. Curr. Opin. Psychiatry.

[bb0450] Spek V., Cuijpers P., Nyklíček I., Riper H., Keyzer J., Pop V. (2007). Internet-based cognitive behaviour therapy for symptoms of depression and anxiety: a meta-analysis. Psychol. Med..

[bb0455] Stice E., Marti C.N., Shaw H., Jaconis M. (2009). An 8-year longitudinal study of the natural history of threshold, subthreshold, and partial eating disorders from a community sample of adolescents. J. Abnorm. Psychol..

[bb0460] Striegel Weissman R., Rosselli F. (2017). Reducing the burden of suffering from eating disorders: unmet treatment needs, cost of illness, and the quest for cost-effectiveness. Behav. Res. Ther..

[bb0465] Tasca G.A., Gallop R. (2009). Multilevel modeling of longitudinal data for psychotherapy researchers: the basics. Psychother. Res..

[bb0470] Tasca G.A., Illing V., Balfour L., Krysanski V., Demidenko N., Nowakowski J., Bissada H. (2009). Psychometric properties of self-monitoring of eating disorder urges among treatment seeking women: ecological momentary assessment using a daily diary method. Eat. Behav..

[bb0475] Udo T., Grilo C.M. (2018). Prevalence and correlates of DSM-5–defined eating disorders in a nationally representative sample of US adults. Biol. Psychiatry.

[bb0480] Udo T., Grilo C.M. (2019). Psychiatric and medical correlates of DSM-5 eating disorders in a nationally representative sample of adults in the United States. Int. J. Eat. Disord..

[bb0485] Udo T., Grilo C.M. (2020). Physical activity levels and correlates in nationally representative sample of US adults with healthy weight, obesity, and binge-eating disorder. Int. J. Eat. Disord..

[bb0490] Udo T., Bitley S., Grilo C.M. (2019). Suicide attempts in US adults with lifetime DSM-5 eating disorders. BMC Med..

[bb0495] Venkatesh A., Chang A., Green E.A., Randall T., Gallagher R., Wildes J.E., Graham A.K. (2021). Perceived facilitators and barriers to engaging with a digital intervention among those with food insecurity, binge eating, and obesity. Nutrients.

[bb0500] Vollert B., Beintner I., Musiat P., Gordon G., Görlich D., Nacke B., Grant N. (2019). Using internet-based self-help to bridge waiting time for face-to-face outpatient treatment for bulimia nervosa, binge eating disorder and related disorders: study protocol of a randomized controlled trial. Internet Interv..

[bb9040] Watson D., Clark L.A. (1994). The PANAS-X: manual for the positive and negative affect schedule-expanded form.

[bb9050] Wilson G.T., Zandberg L.J. (2012). Cognitive–behavioral guided self-help for eating disorders: effectiveness and scalability. Clin. Psychol. Rev..

[bb0510] Wyssen A., Meyer A.H., Messerli-Bürgy N., Forrer F., Vanhulst P., Lalanne D., Munsch S. (2021). BED-online: Acceptance and efficacy of an internet-based treatment for binge-eating disorder: A randomized clinical trial including waitlist conditions. Eur. Eat. Disord. Rev..

[bb0515] de Zwaan M., Herpertz S., Zipfel S., Svaldi J., Friederich H.-C., Schmidt F., Hilbert A. (2017). Effect of internet-based guided self-help vs individual face-to-face treatment on full or subsyndromal binge eating disorder in overweight or obese patients: the INTERBED randomized clinical trial. JAMA Psychiatry.

