# Supplementary material for: Integrating a web-based intervention into routine care of binge-eating disorder: Study protocol for a randomized controlled trial
Source: Internet Interv. 2022 Feb 21;28:100514. doi: 10.1016/j.invent.2022.100514 (PMC8907668; doi:10.1016/j.invent.2022.100514)
Supplement: Supplementary file 1 — Supplementary material to the randomized controlled trial of a web-based intervention for binge eating disorder. [file mmc1.docx]

Supplementary Material

for

**Integrating a web-based intervention into routine care of binge-eating disorder:**

**Study protocol for a randomized controlled trial**

Pruessner, Luise^1^;

Hartmann, Steffen^1^;

Rubel, Julian A.^2^;

Lalk, Christopher^2^;

Barnow, Sven^1^;

Timm, Christina^1^

^1^ Department of Psychology, Heidelberg University, Hauptstr. 47-51, 69117 Heidelberg

^2^ Psychotherapy Research Unit, University of Giessen, Otto-Behaghel-Straße 10, 35394 Giessen

**PICO study overview**

Table S1

*Overview of the study based on the PICO framework*

| Criteria | Assessments |
| --- | --- |
| **P:**  Population | *Inclusion criteria:*   1. age between 18 and 65 years 2. sufficient German language skills (C1) 3. a smartphone with permanent internet access during the study period 4. meeting the diagnostic criteria for BED according to the Diagnostic- and Statistical Manual of Mental Disorders (DSM-5)   *Exclusion criteria:*   1. Body Mass Index (BMI) below 18.5 2. current psychotherapy or pharmacotherapy for eating disorders 3. anorexia nervosa or bulimia nervosa 4. comorbid bipolar disorder or psychotic disorders 5. acute substance dependence 6. current severe depressive episode 7. acute suicidality |
| **I:**  Intervention | - 12 weeks of a web-based intervention for BED (*Selfapy)* |
| **C:**  Control | - 12 weeks of waiting time |
| **O:**  Outcome | *Primary Confirmatory*   - Number of binge eating episodes (EDE-Q)   *Secondary Confirmatory*   - Global eating psychopathology (EDE-Q) - Weekly Binges Questionnaire (WBQ) - Clinical impairment (CIA) - Well-being (WHO-5) - Work capacity (*i*PCQ)   *Secondary Exploratory*   - Comorbid depression (PHQ-9) - Comorbid anxiety (GAD-7) - Self-esteem (RSES) - Difficulties in emotion regulation (DERS) - Emotion regulation frequencies (HFERST) - Everyday eating disorder symptoms and emotion regulation (EMA) |

**R script of the confirmatory analyses**

Supplement S2

*Script for the confirmatory data analyses*

**#COMPLETER- analysis---**

*#reshape dataset:*

df_long = reshape(data = df,

idvar = "ID",

direction = "long",

varying = list(c("Y1","Y2","Y3")),

timevar = "TIME",

times = paste(0:2),

v.names = "Y")

df_long$TIME = as.numeric(df_long$TIME)

*#calculation:*

mod0 <- lmer(Y ~ 1 + (1|ID), data= df_long)

mod1 <- lmer(Y ~ TIME + (1|ID), data= df_long)

mod2 <- lmer(Y ~ TIME*GROUP + (1|ID), data= df_long)

anova(mod0, mod1, mod2)

**#LOCF imputation ----**

LOCF_df = df

LOCF_df$Y2[is.na(LOCF_df$Y2)] <- LOCF_df$Y1[is.na(LOCF_df$Y2)]

LOCF_df$Y3[is.na(LOCF_df$Y3)] <- LOCF_df$Y2[is.na(LOCF_df$Y3)]

*#reshape dataset:*

LOCF_df_long = reshape(data = LOCF_df,

idvar = "ID",

direction = "long",

varying = c("Y1", "Y2","Y3"),

timevar = "TIME",

times = paste(0:2),

v.names = "Y")

LOCF_df_long$TIME = as.numeric(LOCF_df_long$TIME)

*#calculation:*

mod0.locf <- lmer(Y ~ 1 + (1|ID), data= LOCF_df_long)

mod1.locf <- lmer(Y ~ TIME + (1|ID), data= LOCF_df_long)

mod2.locf <- lmer(Y ~ TIME*GROUP + (1|ID), data= LOCF_df_long)

anova(mod0.locf, mod1.locf, mod2.locf)

**#MICE imputation**

*# imputation based on control group data:*

ignore_vector <- df$GROUP

ignore_vector <- as.logical(ignore_vector)

*#MICE imputation, 5 imputations, ignore intervention group:*

imp_mice <- mice(data = df, m = 5, ignore = ignore_vector)

*#transform mice object into R list:*

df_mice <- as.list(1:5)

for(i in 1:5){

df_mice[[i]] <- mice::complete(imp_mice, action=i) }

*#transform to long format:*

df_mice_long <- lapply(df_mice, pivot_longer, cols = c("Y1","Y2","Y3"), names_to = "times", values_to = "Y")

i = 1

while (i < 6)

{

df_mice_long[[i]]$TIME <- 0

df_mice_long[[i]]$TIME[df_mice_long[[i]]$times == "Y2"] <- 1

df_mice_long[[i]]$TIME[df_mice_long[[i]]$times == "Y3"] <- 2

df_mice_long[[i]]$TIME = as.numeric(df_mice_long[[i]]$TIME)

i = i + 1

}

*#calculation:*

mod0.mice <- lmer(Y ~ 1 + (1|ID), data= df_mice_long)

mod1.mice <- lmer(Y ~ TIME + (1|ID), data= df_mice _long)

mod2.mice <- lmer(Y ~ TIME*GROUP + (1|ID), data= df_mice _long)

anova(mod0.mice, mod1.mice, mod2.mice)

**#calculation of effect size**

*#extract effect:*

b3 = as.numeric(fixef(model)['TIME:GROUP'])

*#calculation of pooledSD:*

SD_raw_pre_t <- filter(df, GROUP == "1") %>% summarise(s = sd(Y1)) %>% pull() # treatment baseline SD

SD_raw_pre_c <- filter(df, GROUP == "0") %>% summarise(s = sd(Y1)) %>% pull() # control baseline SD

*#insert group size:*

n_t <- 76

n_c <- 76

SD = sqrt(((n_t - 1) * SD_raw_pre_c^2 + (n_c - 1) * SD_raw_pre_t^2) / (n_t + n_c - 2))

*#Cohen's d:*

time = 2

d = b3*2/SD

*#CI:*

b_SE = summary(model)$coef[4, 2, drop = FALSE][1,1]

LCL_b = b3 - (1.96*b_SE)

UCL_b = b3 + (1.96*b_SE)

LCL_d = (LCL_b*2)/SD

UCL_d = (UCL_b*2)/SD


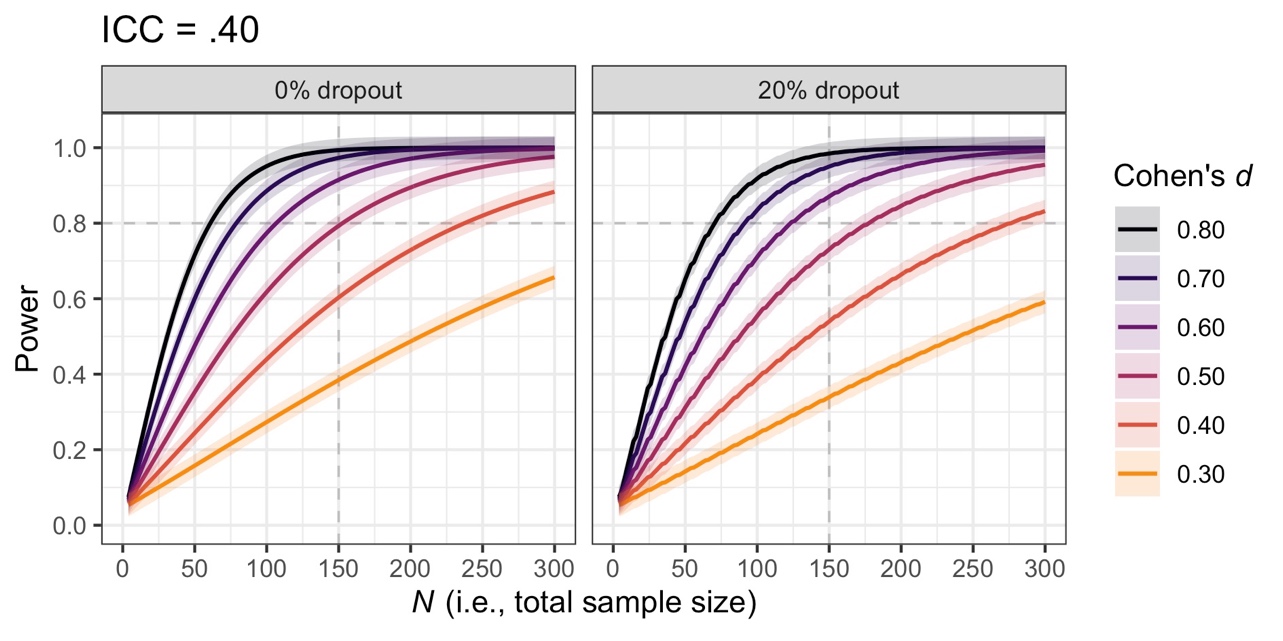
**Power Analyses**


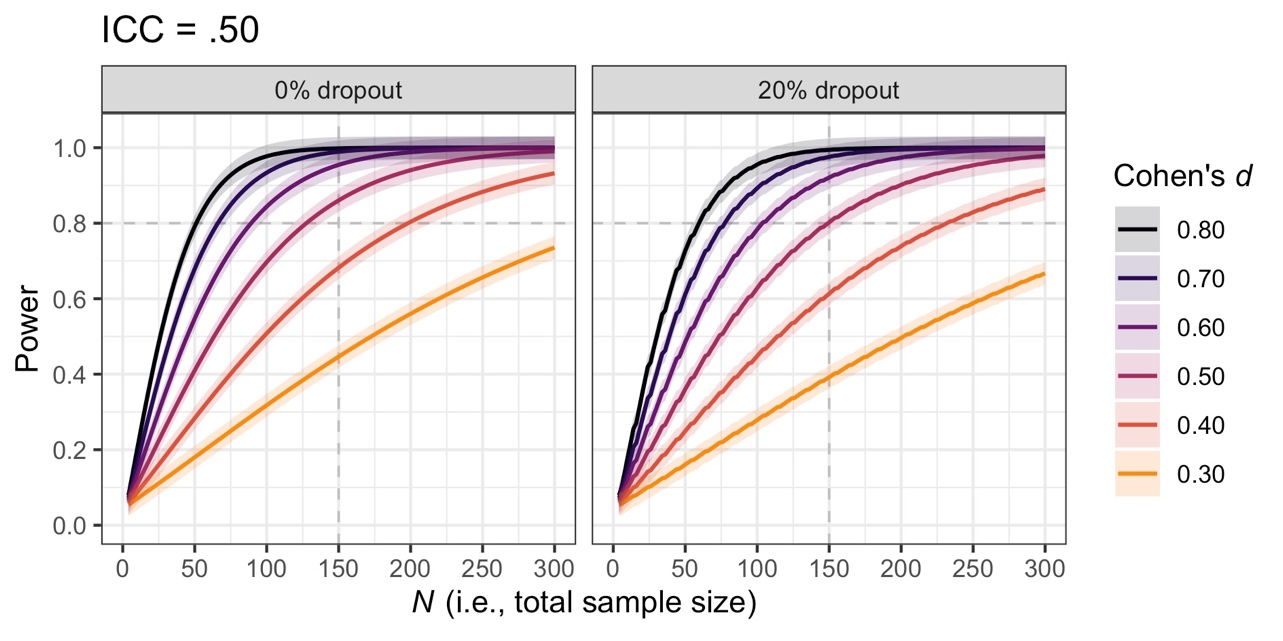


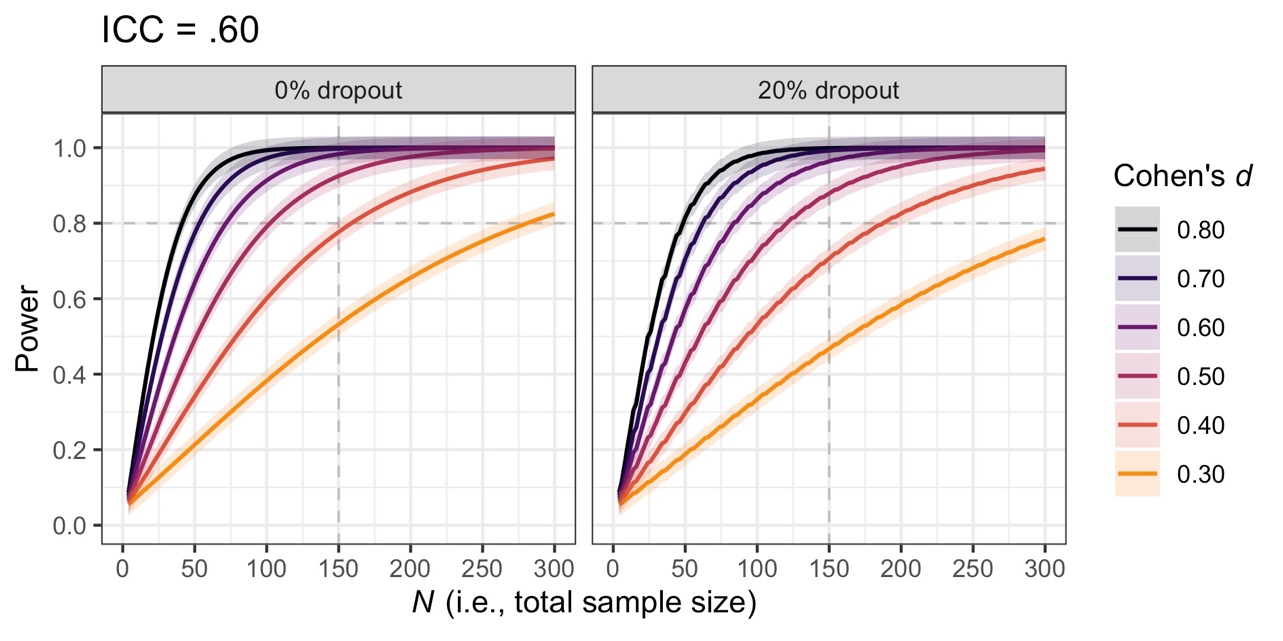


*Figure S3*. Power analyses are based on the following parameters: α = .05, power = .80, number of groups = 2, number of measurements = 3.
